# The Dangers of Involving Children as Family Caregivers of Palliative Home-Based-Care to Advanced HIV/AIDS Patients

**DOI:** 10.4103/0973-1075.73641

**Published:** 2010

**Authors:** Simon Kangethe

**Affiliations:** Department of Adult Education, Centre for Continuous Education, University of Botswana, P/B UB 00707, Gaborone, Botswana

**Keywords:** Care giving, Community home based care program, HIV/AIDS, Palliative minor caregivers, Perfidy

## Abstract

The aim of this research paper is to explore the dangers of involving children as family caregivers of palliative care and home-based-care to advanced HIV/AIDS patients, while its objective is to discuss the dangers or perfidiousness that minors especially the girl children face as they handle care giving of advanced HIV/AIDS patients. The article has relied on eclectic data sources. The research has foundminors disadvantaged by the following: being engulfed by fear and denied rights through care giving; being emotionally and physiologically overwhelmed; being oppressed and suppressed by caring duties; being at risk of contracting HIV/AIDS; and having their education compromised by care giving. The paper recommends: (1) strengthening and emphasizing on children’s rights; (2) maintaining gender balance in care giving; (3) implementation and domestication of the United Nations conventions on the rights of children; (4) community awareness on equal gender co participation in care giving; (5) and fostering realization that relying on child care giving is a negative score in fulfilling global Millennium Development Goals.

## OPERATIONAL DEFINITION

Perfidy is a bad and undesirable state of affairs.

Minors are usually children below 18 years usually at their teenage or adolescent stages. The word minors and children will be used interchangeably.

### Statement of the problem

The issue of having the minors handle palliative care giving tasks to advanced HIV/AIDS patients is an outcry especially from the international human rights bodies. These bodies are advocating for the rights of children and minors against being subjected to tasks that will stress their psychological and emotional capacity. It is also against the international labor organizations (ILO) and conventions on the rights of children.[1,2] This is also a bottleneck facing many developing countries as they struggle toward achieving their national goals as regards the rights of children as well as the Global Millennium Development Goals.[3] This paper, therefore, forms a discussion forum on the gaps and contributory factors burdening the minors especially the girl child as opposed to the boy child. It would also be a robust advocacy forum indicating and reinforcing strategies for a paradigm shift of the prevalent factors that contribute to this quagmire. The need for a healthy and balanced gender equality and equity in palliative care giving between boys and girls, and men and women is critical.[4,5]

## BACKGROUND AND INTRODUCTION

Care giving of the sick persons carried out by the minors especially the girl children in most societies of the world perceptibly emanates from cultural beliefs, norms and tradition’s interpretations that girls need to occupationally follow the footsteps of their mothers and grandchildren.[6,7] This is also richly bolstered by most societies’ patriarchal thinking and ideologies.[4,5] This has therefore seen the girl children coming in to fill care giving gaps when the mothers and the grandmothers are not in a position, either due to poverty, sickness, or old age, or family disintegration due to factors such as divorce or separation of their parents. Increasingly, the global community is grappling to fight the horrendous and pinching effects of HIV/AIDS against the backdrop of inadequate resources. Care giving carried by the girl children is one such scenario that HIV/AIDS has immensely ushered in the developing world.[8] At the same time, the rights of those living with the virus have been emphasized, as well as respecting the rights of the children against holding tasks which their social, emotional and psychological capacity cannot hold.[1,2] Ironically and unfortunately, many children have found themselves being the economic agents of their households, heads of households, and carers of their sick parents, brothers and sisters. This state of children has not respected their capacities and energies to adequately carry these tasks, and the life building opportunity such as acquisition to education that care giving compromises. In many care giving scenarios and contexts, child caregivers demonstrate recognizable physical and emotional responses to their situation. These can include, but are not limited to:

1. changes in social behaviors,
2. decline in school performance,
3. decreased participation in previously enjoyable activities,
4. mood disturbances,
5. increased fatigue,
6. personality changes, and
7. self-isolation.[9]

Changes in social behaviors can be seen in the way they interact with both adults and other children. Some use more adult language, engaging adults in social situations rather than persons of their own age, while others appear to regress or demonstrate attention-seeking behaviors such as baby talking, excessive crying or thrill seeking. School performance changes can result from preoccupation or worry about the ill or disabled person, though this is generally more prevalent at the beginning of the changes at home than when the situation is long-term. Behaviors which are disruptive in social situations affect school, as well, and the child may talk in class, become tearful, or frequently become emotionally disturbed making coping with his/her age mates an uphill task.[1,10]

Minor caregivers’ normal child growth and development may take an abnormal course which may spill over to their adult life. Mood swings are commonly evident in some youngsters. A sense of loss of control, fear, or guilt that they may have been the cause of the illness (if they have suffered a significant loss), can manifest themselves in very strong feelings. Incidents that would not have warranted even a mild response can become gigantic and the focus of these strong emotions may result in verbalized and sometimes displaced anger. And, as children have generally less sophisticated ways in which to communicate their feelings, they may express them as behaviors.[10,11] Research on child care giving in both developed and developing nations suggests that minor care giving can be located along a ‘care giving continuum’ and that young carers, globally, have much in common irrespective of where they live or how developed are their national welfare systems. The research recommends that there is a need in all countries for minor caregivers to be recognized, identified, analyzed and supported as a distinct group of ‘vulnerable children.”[12] Considering this care giving environment of the minors, interrogating it with the hope of changing the situation or augmenting it positively is pivotal to influencing the fulfillment of the United Nations Millennium Development Goals (MDGs) that envisage to see a healthy, well gendered society especially children; and Vision 2016 aspirations that envisage to see a well gendered, healthy, educated nation.[3,13]

The plight of involving minors especially to carry out adult tasks is also contained in a February 1997 address to the world Economic Forum in which Mandela, former South African President pointed to the challenge of palliative care giving that minors face as the epidemic takes toll. He indicated that HIV/AIDS poses the greatest threat to Africa’s effort to achieve its full potential, putting in the balance the future of nations; kills those on whom the society relies on to grow the crops, work in the mines and factories, run the schools, hospitals and those that govern countries. HIV/AIDS, therefore, creates new pockets of poverty when parents and breadwinners die and children leave school earlier to support the remaining children.[2,11] These minors sometimes become the caregivers to offer care to the HIV/AIDS parents and close relatives and sometimes take over as heads of households. In this context, the care of HIV/AIDS clients by the minors, though unfortunate as it sacrifices their education and their future, is ironically an important contribution to palliative care giving.

The rights of children or for the interests of this article, minors, is richly grounded and emphasized by the United Nations Conventions on the Rights of Children. By 1999, all countries (except Somalia and United States) including Botswana had ratified the convention. The convention has borrowed and applied the human rights espoused and enshrined in the Universal Declaration that indicates that childhood is entitled to special care and assistance.[1,2] To achieve these rights call for nurturing a conducive environment of happiness, love and understanding that fosters the growth and the well being of children. These factors are also reinforced by the Geneva Declaration on the rights of the child of 1924; Declaration of the rights of the child adopted by the General Assembly on 20^th^ November 1959;International Covenant on Civil and Political Rights (in particular in articles 23 and 24); International Covenant on Economic, Social and Cultural Rights (in particular article 10); and statutes and relevant instruments of specialized agencies and international organizations concerned with the welfare of children. All in all, care giving left in the shoulders of the minors is geometrically and diametrically opposed to these international covenants. It is crystal clear that the rights of the child are bluntly being sacrificed by the incidences of the HIV/AIDS pandemic. It is therefore timely and relevant to challenge most countries to domesticate these conventions. This is because the treatment meted against the children through imposing care giving load on their shoulders indicates little commitment, understanding, or poor operationalization of these conventions[2]

### Perfidious nature of care giving by the minors

#### Fear and denied rights of the minors through care giving

Minors are usually not psychologically and emotionally in a position to handle HIV/AIDS clients or even withstand the social environment and conditions inherent with people living with virus. This is because care giving is socially, emotionally and psychologically draining, driving the actors into a state of dilemma, desperation and sometimes depression.[10,14] The care giving load subjected to the minors leaves them desperate, fatigued, despondent and in a confused state of mind. Developing state of insomnia, dementia, and mental exhaustion are known characteristics inherent with care giving of especially persons living with HIV/AIDS.[15,16] Article 32 of the Convention on the Rights of the Child recognizes the right of the child to be protected from economic exploitation, and from performing any work that is likely to be hazardous or to interfere with the child’s health, physical, mental, spiritual, moral or social development. It is stark naked reality that care giving offered by the minors contravenes all these contents of the convention.[1,2]

Researchers have expressed the sorry state of affairs as minor children are confronted with the quagmire of facing HIV/AIDS situations. For example in their research, Clacherty and Associates[17] indicate the traumatizing phenomenon of a boy whose mother was living with HIV/AIDs. The boy expressed his fear associated with the sickness of his family by qualitatively saying that:

“*When my mother is sick, we become sad with my sister. I think I am afraid. We cry sometimes. I am afraid my grandmother is also sick”.*

Such is an expression of unprocessed emotional burden especially confronting the mind of a minor whose capacity to process the dilemma is limited.[18] This also indicates a state of grief that the boy and the family members need to be prepared or helped to process. According to Uys and Cameron,[10] unprocessed grief could lead to cynicism, dementia, insomnia, paranoia, confusion and mental disorderliness. These are factors that could easily lead to heart attack of even the minors.

#### Girl children are oppressed and suppressed by caring duties

According to Smart,[19] the reality of the HIV/AIDS in the family is that children especially the girl children are caring for the sick and assuming adult responsibilities before they are ready to do so. They leave school earlier, marry earlier, enter into the labor market earlier, and are frequently sexually exploited.[20] Smart[19] calls this parentification, referring to the process of creating a parent out of a child in order to care for a parent or siblings. According to a score of psychologists, this has an impact toward their physical, social, and emotional development and could manifest and pose a life challenge in their future especially when it comes to confidence, assertiveness and relating with opposite sex parties[21,22] This is associated with social isolation. Younger children not only assume responsibility for more complex household chores, but are also deprived of their nurturing previously received from their ill parents. This according to the United Nations’ Conventions on labor amounts to exploitation.[2,19] According to article 19 of the convention on the rights of the child, children need to be protected from all forms of maltreatment or exploitation including sexual abuse. This author feels that palliative care giving bestowed upon the minors is maltreatment as it vexes and tickles their emotional, social and psychological equilibrium, and therefore contradicts this convention.[1]

Reality on the ground indicates that some children whose parents have died of HIV/AIDS are forced to enter into care giving without any choice. They usually get into the service physically, psychologically and emotionally unprepared, and therefore find themselves victims of trauma, dilemma, guilt and grief.[18] In many care contexts today, child headed household phenomenon is increasing commensurately with burgeoning cases of HIV/AIDS care especially in the developing world where HIV/AIDS statistics continue to take toll.[23] Smart[19] indicates the following challenges that child headed households experience:

- poverty;
- lack of supervision;
- stunting and hunger;
- education failure;
- lack of adequate medical care;
- psychological problems;
- disruption of normal childhood and adolescence;
- exploitation;
- early marriage; discrimination;
- poor housing;
- and child labor

The above characteristics demonstrated by the environment of child headed environment makes it crystal clear that the contents of the Declaration of the Rights of the child still need to be emphasized, strongly applied, domesticated and operationalized. The contents from the bullets indicate information that is geometrically and diametrically opposed to the declaration that “the child by reason of its physical and mental immaturity needs special safeguards and care, including appropriate legal protection, before as well as after birth”.[1,2]

#### Children are missing education due to care giving

Besides increased care rendered by minors especially the girl children due to increased human resource gap, poverty and other life debilitating aspects embedded in culture and patriarchy dynamics, HIV/AIDS phenomenon continue to impact negatively on the school chances of especially the girl children. This is because culture and socialization continue to dictate and demarcate the role of the girl child in taking the role of the mother and grandmothers especially in care tasks once the latter are not able to do so due to death, incapacitation, or other social reasons. Losing chances of education is a worrisome state of affairs as it impacts negatively toward the realization of Millennium Development Goals especially goal 2 that aspires to see all countries achieve universal primary education by the year 2015,[3] while it also suppresses Vision 2016 goal in Botswana that envisages seeing the country becoming a soundly educated and informed nation.[13] Care giving, therefore, has been found to affect schooling for the girls. In a survey done in South Africa, for example, over 40% of the affected households reported that the main caregiver had taken time off work or school to take care of an AIDS patient. Girls leaving school permanently could be a possibility.[24]

In Botswana, girls leaving school can be a threat to its perfect Gender Parity Index (GPI) that the country has been healthily experiencing. Gender Parity Index (GPI) measures the primary school enrolment of the girl children compared to boys. While many countries in developing world’s GPI is less than one, that of Botswana and other countries such as Namibia is perfect, or indicated by score one.[25] According to Article 28 of the Convention on the Rights of the Child (CRC), the child has the right to education and all countries have the obligation to:

- ensure that primary education is free and compulsory;
- encourage different forms of secondary education accessible to every child and to make higher education available to all on the basis of capacity.

Care giving vested in the hands of the minors runs contrary to the will of the contents of this convention and also against the fulfillment of universal primary education by the year 2015.[26,27]

To ground the arguments with empirical examples, this author’s research in 2005/2006 in Kanye village of Botswana revealed how gender dynamics were likely to sacrifice a girl’s school attendance in order to do care while boys were at large not attending school. This therefore indicates how retrogressive some of these gender dynamics embedded in cultural forces and patriarchy are. This calls for a serious gender autopsy, analysis and transformation to ensure that both genders co–participate in sharing the load presented by HIV/AIDS scourge[15]

The fact that teachers may miss classes as they have to attend to their beloved ones who may desperately need their care exacerbates the school situation. Issues of increased burial ceremonies that the teachers as members of the society have to attend in Botswana, for instance, may also add complexes associated with HIV/AIDS that may offset their psychological, emotional and physical strength to adequately be productive at school.[24]

#### Minors at risk of contracting HIV/AIDS

Due to lack of knowledge, experience and lowered age, children offering care face increasing risk of contagion as they come into contact with diseased skin of their loved ones, possibly when they have no protective clothing. These children may not have had the opportunity to be trained in care giving, nor would they have had the opportunity to have learnt of the prevention issues adequately. In his 2005-2006 research, this author learnt from his caregiver research participants that there were girl children who were not registered in the community home-based care register, yet involved in palliative care giving. Since some caregiver research participants suspected some of them were succumbing to HIV/AIDS due to their care giving preoccupation, the girl children who were doing care giving must be at a higher risk due to their age and in exposure.[15,28] This is serious as those trying to reinforce care and support become the victims of the disease. This author’s findings in his 2005–2006 research in Kanye on the risks and hazardous effects associated with handling and disposal of clinical waste could immensely be detrimental to children caregivers.[28] According to article 24 of the convention of the rights of children, a child is entitled to the highest standard of health, medical care and provision of primary preventative health care and public health education. No child should be deprived of the access to effective health services. With care giving of the minors, the operationalization, application and awareness of these contents of the convention may not be achieved.

### Strategic recommendations

#### Strengthen and emphasize on children’s rights

All stakeholders including the government, NGOs, private sector and individuals need to support and own global consensus and mobilizations on children’s rights especially the need for their holistic protection. This is the only way to keep abreast with the children’s human rights. This should go with the realization of the impact of HIV/AIDS in producing orphans. The need to consider the guardians and the communities in which these orphans live with is also critical.[29] Having the governments emphasize on holding the children’s national days such as the Day of the African Child (commemorated every 16^th^ of every June) by all the organizations can make the message spread faster.

#### Gender balance in care giving is critical

Many care giving environments especially in developing countries tax the girl children while the boy children are excused from care giving by culture and socialization. In his research in Kanye in 2005-2006, Kang’ethe[15] indicates episodes of some girl children who would leave school to take care of their ailing parents while there were elderly boy children who may not be at school to handle the task. A paradigm shift is necessary to dilute the cultural and patriarchal arrangements that position girls in the niche of exploitation through care giving. The two genders should co participate in care giving. Gender differentials in most developing countries is entrenched and influenced by patriarchy and cultural inhibitions making clear-cut role differentiation and demarcation. Gender mainstreaming and campaign directed to all the learning institutions is critical. This is to prepare both boys and girls to be gender sensitive, neutral and responsive.

#### Implementation of United Nations Conventions on the Rights of Children

Domestication and implementation of the United Nations Conventions on the rights of Children (UNCRC) contents is critical in relieving children out of the care giving quagmire.[2] Though many countries of the world have signed these conventions, domestication and implementation of the articles remains theoretical, lacking practical and empirical results. If well implemented, the UNCRC will ensure its tenets and aspirations do positively influence the countries. The CRC guarantees the rights of children to:

- protection from maltreatment,
- neglect,
- all forms of exploitation,
- provision of food,
- healthcare,
- education,
- social security,
- and participation in all matters concerning them.

Domestication and operationalization of the conventions will ensure that children’s physical, emotional, societal and intellectual needs are adequately met. This will ensure they enjoy life, develop their full potential, and develop to be fully participating and contributing adults. If these needs remain unmet, or inadequately unmet, the development may become stunted or distorted[21,22]

#### Dilute cultures and gender dynamics oppressing the girl child

UNAIDS[24] indicates the vulnerability in which girls and young women find themselves in. This is because social norms impose a dangerous ignorance on girls and young women who are expected to know little about sex and sexuality at large. This lack of knowledge magnifies their risk of HIV infection. A survey in Cameron, Lesotho, Mali, Senegal and Vietnam to girls and young women of ages 15-24 indicated they did not know three HIV prevention methods: while in Moldova, Ukraine and Uzbekistan, 80% of this group lacked the same knowledge. The cultural dynamics that solidly position girls and women to a niche of ignorance as far as prevention is concerned need to be obliterated. The cultural dilution may possibly offer an alternative to the place of girls in care giving.[24]

#### Community awareness on co participating in care giving

Community mobilization campaigns are necessary to indicate to families, communities, societies and governments in general that they should not rely on women’s fortitude and resilience to provide sustainable care giving safety nets. Other players need to come on board, with men taking the lead.[30] It is also recommendable that AIDS home care programs be extended beyond medical and nursing care to include counseling, food assistance, welfare support, schooling subsidies and income opportunities that would benefit households. This is likely to come out with human resource package to relieve the girl child back to school.[24]

#### Realization that relying on child care giving is a negative score to fulfilling Millennium Development Goals

Probably at a global perspective, leaving children to take care of the HIV/AIDS clients as a desperate move makes a negative score towards realizing goal 6 of the MGD that intends to halt and reverse the spread of the HIV/AIDS by the year 2015[27,31] Failure to meet the goal 6 of halting the spread of HIV/AIDS will also adversely affect the world’s chances of progress on the other MDGs, such as reduction of poverty; provision of universal primary education; and reduction of child mortality and empowerment of maternal deaths.[31] At a national level in Botswana, this poses a bottleneck to the realization of the country’s MDG and vision 2016 goals and aspirations of an AIDS free generation.[4,13]

## CONCLUSION

Having incidences of care giving being vested into the hands of the minors is a defeatist syndrome in the campaign against HIV/AIDS and attainment of Millennium Development Goals. The minors’ social, psychological, emotional and intellectual capacity has not matured to adequately handle the dilemmas and complexities presented by HIV/AIDS. The government and local leaders in Botswana need to realize the dangers that the exercise poses to the girl children, especially of sacrificing her education acquisition opportunity. A paradigm shift to possibly involve men into care giving is likely to offset and obliterate factors that impose the girl children as caregivers in the place of their mothers and grandmothers. Having care giving institutionalized homes or day care centers could possibly create vents for the girl children to escape care giving tasks.
